# Supplementary material for: Exploring medical error taxonomies and human factors in simulation-based healthcare education
Source: PLoS One. 2025 Jan 17;20(1):e0317128. doi: 10.1371/journal.pone.0317128 (PMC11741583; doi:10.1371/journal.pone.0317128)
Supplement: S1 Appendix — (DOCX) [file pone.0317128.s001.docx]

**Appendix A. Questionnaire employed for data collection in this study.**

To err is human; people will continue to make mistakes, and medicine is no exception. However, making mistakes in a simulation center within a safe learning environment is certainly better than in clinical practice. To speak the same language when it comes to the theory of errors and to be able to properly understand, describe, and evaluate mistakes, it is necessary to first name and categorize them. Your responses will remain anonymous. The research team will not identify you in any report based on the information provided in this questionnaire. Upon submitting the questionnaire, you agree that your comments will follow anonymous citations in publications related to this research. Therefore, I kindly ask you to complete it, which should take no more than five minutes. Thank you very much for your cooperation, and I wish you much success in your professional and personal lives.

**Q1. What types of medical errors do you believe occur in everyday practice?**

Rate 1 (most frequent) to 5 (least frequent) using each number only once.

_ Slips: action-related mistakes, “autopilot”

_ Lapses: memory-based mistakes, where we forget what we planned to do, or in what order we intended to do it

_ Rule-based mistakes: misapplication of a good rule or failure to apply

a good rule, usually because we are not aware of its contraindications or

application of a bad rule

_ Knowledge-based mistake: related to any type of knowledge, general, specific,

or expert

_ Violation: the act knowingly incurs risk

**Q2. Have you noticed any medical error(s) during today´s course?**

If yes, please mark which one(s) (multiple answers possible).

□ Slips

□ Lapses

□ Rule-based mistake

□ Knowledge-based mistake

□ Violation

**Q3. Please describe this error/these errors briefly:**

**Q4. What types of medical errors do you believe occur in everyday practice?**

Rate 1 (most frequent) to 5 (least frequent) using each number only once.

_ **Sloth:** not doing what the effort required or perceiving as an inadequate reward

_**Fixation:** particular diagnosis or analysis is firmly held onto despite

evidence against it, confirmation bias

_ **Communication breakdown:** paramount information is not released or does not reach its destination at the right time

_ **Poor team working team:** members out of their abilities, lack of leadership

_ **Playing the odds:** failure to understand the fundamental rules of probability, preferring despite a well-known diagnosis

_ **Bravado:** clinicians work beyond their competence or lack adequate

supervision

_ **Ignorance:** unconscious incompetence, lack of knowledge

_ **Mis-triage:** over – or underestimation of the seriousness of a situation and lack of prioritization

_ **Lack of skill:** lack of teaching or practice

_ **System error:** unnecessary decision-making steps, multiple distractions, lack of checklists, lack of policies

**Q5. Have you noticed any medical error(s) during today´s course?**

□ Sloth

□ Fixation

□ Communication breakdown

□ Poor team working team

□ Playing the odds

□ Bravado

□ Ignorance

□ Mis-triage

□ Lack of skill

□ System error

**Q6. Please describe this error/these errors briefly:**
