# Supplementary material for: Exploring medical error taxonomies and human factors in simulation-based healthcare education
Source: PLoS One. 2025 Jan 17;20(1):e0317128. doi: 10.1371/journal.pone.0317128 (PMC11741583; doi:10.1371/journal.pone.0317128)
Supplement: Attachment — Submitted filename: PONE-D-24-13204.pdf [file pone.0317128.s002.pdf]

PLOS ONE

Exploring Medical Error Taxonomies and Human Factors in Simulation-based Healthcare Education

--Manuscript Draft--

**Manuscript Number:**	PONE-D-24-13204
**Article Type:**	Research Article
**Full Title:**	Exploring Medical Error Taxonomies and Human Factors in Simulation-based Healthcare Education
**Short Title:**	Redefining Medical Error Taxonomies for Simulation Training
**Corresponding Author:**	Daniel Schwarz<br>Masaryk University Faculty of Medicine: Masarykova univerzita Lekarska fakulta<br>Brno, CZECH REPUBLIC
**Keywords:**	patient safety; Medical Error; taxonomy; classification
**Abstract:**	This research delves into the effects of medical errors on patient safety, stressing the need for a suitable classification system to effectively handle such errors. Despite numerous existing taxonomies for medical errors, none appear to cater specifically to simulation-based educational settings in healthcare. Rather than creating a new system, the researchers propose merging existing classifications to enhance their applicability in simulation training. To comprehend the complexities better, they examined two classifications – a generic one by Reason and an education-specific one by Round. After comparing these models, they questioned which would be more useful in a simulation-based education scenario. While a generic classification can encompass a wide range of situations, a domain-centric classification could be more relatable and easily understood by healthcare professionals – potentially boosting error reporting rates. Seven strong links between reviewed medical error classifications were identified. This led to the proposal of an interpretation and possible training approach in simulation settings with a focus on these connections. Such an approach could enhance understanding and training concerning identified medical errors. Significant correlations were also found between different error classifications, indicating that addressing one error type could positively influence others. Finally, the study underlines the importance of simulation-based methods in healthcare, positioning them as an invaluable tool for medical error research, management, and overall performance improvement.
**Order of Authors:**	Tamara Skrisovska, MD, DESAIC
	Daniel Schwarz, PhD
	Martina Kosinova, PhD
	Petr Stourac, MD, PhD, FESAIC
**Additional Information:**	
**Question**	**Response**
**Financial Disclosure**<br><br>Enter a financial disclosure statement that describes the sources of funding for the work included in this submission. Review the submission guidelines for detailed requirements. View published research articles from *PLOS ONE* for specific examples.<br><br>This statement is required for submission	Yes

and **will appear in the published article** if the submission is accepted. Please make sure it is accurate.<br><br>**Funded studies**<br>Enter a statement with the following details:<br>• Initials of the authors who received each award<br>• Grant numbers awarded to each author<br>• The full name of each funder<br>• URL of each funder website<br>• Did the sponsors or funders play any role in the study design, data collection and analysis, decision to publish, or preparation of the manuscript?<br><br>Did you receive funding for this work?	
Please add funding details.<br>as follow-up to "**Financial Disclosure**<br><br>Enter a financial disclosure statement that describes the sources of funding for the work included in this submission. Review the submission guidelines for detailed requirements. View published research articles from *PLOS ONE* for specific examples.<br><br>This statement is required for submission and **will appear in the published article** if the submission is accepted. Please make sure it is accurate.<br><br>**Funded studies**<br>Enter a statement with the following details:<br>• Initials of the authors who received each award<br>• Grant numbers awarded to each author<br>• The full name of each funder<br>• URL of each funder website<br>• Did the sponsors or funders play any role in the study design, data collection and analysis, decision to publish, or preparation of the manuscript?<br><br>Did you receive funding for this work?"	This research was partially supported by the Specific University Research grant provided to Masaryk University by the Ministry of Education of the Czech Republic (MUNI/A/1595/2023, MUNI/A/1551/2023) and also in part supported by the Ministry of Health of the Czech Republic (FNBr, 65269705).

Powered by Editorial Manager® and ProduXion Manager® from Aries Systems Corporation

Please select the country of your main research funder (please select carefully as in some cases this is used in fee calculation).<br>as follow-up to "**Financial Disclosure**<br><br>Enter a financial disclosure statement that describes the sources of funding for the work included in this submission. Review the submission guidelines for detailed requirements. View published research articles from *PLOS ONE* for specific examples.<br><br>This statement is required for submission and **will appear in the published article** if the submission is accepted. Please make sure it is accurate.<br><br>**Funded studies**<br>Enter a statement with the following details:<br>• Initials of the authors who received each award<br>• Grant numbers awarded to each author<br>• The full name of each funder<br>• URL of each funder website<br>• Did the sponsors or funders play any role in the study design, data collection and analysis, decision to publish, or preparation of the manuscript?<br><br>Did you receive funding for this work?"	CZECH REPUBLIC - CZ
**Competing Interests**<br><br>Use the instructions below to enter a competing interest statement for this submission. On behalf of all authors, disclose any competing interests that could be perceived to bias this work—acknowledging all financial support and any other relevant financial or non-financial competing interests.<br><br>This statement is required for submission and **will appear in the published article** if the submission is accepted. Please make sure it is accurate and that any funding	The authors have declared that no competing interests exist.

sources listed in your Funding Information later in the submission form are also declared in your Financial Disclosure statement.<br><br>View published research articles from *PLOS ONE* for specific examples.<br><br>**NO authors have competing interests**<br><br>Enter: *The authors have declared that no competing interests exist.*<br><br>**Authors with competing interests**<br><br>Enter competing interest details beginning with this statement:<br><br>*I have read the journal's policy and the authors of this manuscript have the following competing interests: [insert competing interests here]*<br><br>* typeset	
**Ethics Statement**<br><br>Enter an ethics statement for this submission. This statement is required if the study involved:<br><br>• Human participants<br>• Human specimens or tissue<br>• Vertebrate animals or cephalopods<br>• Vertebrate embryos or tissues<br>• Field research<br><br>Write "N/A" if the submission does not require an ethics statement.<br><br>General guidance is provided below. Consult the submission guidelines for detailed instructions. **Make sure that all information entered here is included in the Methods section of the manuscript.**	Ethical approval was not sought for this study as it focuses on surveying the incidence and frequency of specific medical errors observed during a simulation course and in the medical practice. It is important to note that participation in the survey was entirely voluntary. Furthermore, we ensured complete anonymity of the survey respondents. The study was conducted in accordance with the local legislation and institutional requirements. Informed consent was obtained from all participants as part of the survey introduction.<br><br>Exemption letter has been provided. This letter confirms that the research described in the manuscript is not subject to mandatory ethical review according to the laws of the Czech Republic and the internal regulations of the Faculty of Medicine at Masaryk University. The exemption letter serves to verify that our study complies with relevant ethical guidelines despite being exempt from formal review.

Powered by Editorial Manager® and ProduXion Manager® from Aries Systems Corporation

**Format for specific study types**<br><br>**Human Subject Research (involving human participants and/or tissue)**<br>• Give the name of the institutional review board or ethics committee that approved the study<br>• Include the approval number and/or a statement indicating approval of this research<br>• Indicate the form of consent obtained (written/oral) or the reason that consent was not obtained (e.g. the data were analyzed anonymously)<br><br>**Animal Research (involving vertebrate animals, embryos or tissues)**<br>• Provide the name of the Institutional Animal Care and Use Committee (IACUC) or other relevant ethics board that reviewed the study protocol, and indicate whether they approved this research or granted a formal waiver of ethical approval<br>• Include an approval number if one was obtained<br>• If the study involved *non-human primates*, add *additional details* about animal welfare and steps taken to ameliorate suffering<br>• If anesthesia, euthanasia, or any kind of animal sacrifice is part of the study, include briefly which substances and/or methods were applied<br><br>**Field Research**<br><br>Include the following details if this study involves the collection of plant, animal, or other materials from a natural setting:<br>• Field permit number<br>• Name of the institution or relevant body that granted permission	
**Data Availability**<br><br>Authors are required to make all data underlying the findings described fully available, without restriction, and from the time of publication. PLOS allows rare exceptions to address legal and ethical concerns. See the PLOS Data Policy and FAQ for detailed information.	Yes - all data are fully available without restriction

Powered by Editorial Manager® and ProduXion Manager® from Aries Systems Corporation

A Data Availability Statement describing where the data can be found is required at submission. Your answers to this question constitute the Data Availability Statement and **will be published in the article**, if accepted.<br><br>**Important:** Stating 'data available on request from the author' is not sufficient. If your data are only available upon request, select 'No' for the first question and explain your exceptional situation in the text box.<br><br>Do the authors confirm that all data underlying the findings described in their manuscript are fully available without restriction?	
**Describe where the data may be found in full sentences. If you are copying our sample text, replace any instances of XXX with the appropriate details.**<br><br>• If the data are **held or will be held in a public repository**, include URLs, accession numbers or DOIs. If this information will only be available after acceptance, indicate this by ticking the box below. For example: *All XXX files are available from the XXX database (accession number(s) XXX, XXX.*).<br>• If the data are all contained **within the manuscript and/or Supporting Information files**, enter the following: *All relevant data are within the manuscript and its Supporting Information files.*<br>• If neither of these applies but you are able to provide **details of access elsewhere**, with or without limitations, please do so. For example:<br><br>*Data cannot be shared publicly because of [XXX]. Data are available from the XXX Institutional Data Access / Ethics Committee (contact via XXX) for researchers who meet the criteria for access to confidential data.*<br><br>*The data underlying the results presented in the study are available from (include the name of the third party*	The data underlying the results presented in the study will be made fully available upon acceptance.

and contact information or URL).<br>• This text is appropriate if the data are owned by a third party and authors do not have permission to share the data.<br><br>* typeset	
Additional data availability information:	

Manuscript

Click here to access/download;Manuscript;ExpMedErr-manuscript-after-revision.docx

Exploring Medical Error Taxonomies and Human Factors in Simulation-based Healthcare Education

Tamara Skrisovska[1,2¶], Daniel Schwarz[1¶*], Martina Kosinova[1,2&], Petr Stourac[1,2&]

[1] Department of Simulation Medicine, Faculty of Medicine, Masaryk University, Brno, Czech Republic

[2] Department of Pediatric Anesthesiology and Intensive Care Medicine, University Hospital Brno and Faculty of Medicine, Masaryk University, Brno, Czech Republic

*** Corresponding author**

Email: schwarz@med.muni.cz (DS)

[¶] These authors contributed equally to this work.

[&] These authors also contributed equally to this work.

Abstract

This research delves into the effects of medical errors on patient safety, stressing the need for a suitable classification system to effectively handle such errors. Despite numerous existing taxonomies for medical errors, none appear to cater specifically to simulation-based educational settings in healthcare. Rather than creating a new system, the researchers propose merging existing classifications to enhance their applicability in simulation training. To comprehend the complexities better, they examined two classifications – a generic one by Reason and an education-specific one by Round. After comparing these models, they questioned which would be more useful in a simulation-based education scenario. While a generic classification can encompass a wide range of situations, a domain-centric classification could be more relatable and easily understood by healthcare professionals – potentially boosting error reporting rates. Seven strong links between reviewed medical error classifications were identified. This led to the proposal of an interpretation and possible training approach in simulation settings with a focus on these connections. Such an approach could enhance understanding and training concerning identified medical errors. Significant correlations were also found between different error classifications, indicating that addressing one error type could positively influence others. Finally, the study underlines the importance of simulation-based methods in healthcare, positioning them as an invaluable tool for medical error research, management, and overall performance improvement

1. Introduction

A medical error refers to a preventable unfavorable outcome of medical care, regardless of its visibility or harm to the patient. Data suggest that these occurrences, adverse events caused by unsafe care, are among the top ten – and even top three – leading causes of death worldwide [1, 2]. This conclusion is backed by two pivotal studies from Harvard and Australia [3, 4]. Recognizing the gravity of the situation, the World Health Organization (WHO) has declared patient safety a global health priority through its Global Action on Patient Safety: A Decade of Patient Safety 2020–2030 initiative [5].

This decision affirms that unsafe patient care continues to be a grave worldwide issue, placing significant strain on the healthcare system. The healthcare system's complexity inherently lends itself to mistakes [6, 7]. As of now, the aim is to reduce medical errors, as achieving a completely error-free system is impracticable because of human limitations.

Understanding the nature of medical errors and establishing an effective reporting system is crucial to minimizing them. The foundation of a medical error framework revolves around standardized terminology and classification [8]. Taxonomy elucidates the relationship between items while classification groups them [9]. The WHO World Alliance for Patient Safety proposed standardized terminology in its International Classification of Patient Safety (ICPS) [10]. A taxonomy of medical errors should [illegible]nprehensive, useful for error prediction and reduction, and allow for repeatable documentation [11]. Using consistent language and standard terminology te[illegible]minology reduces, filters and sorts the amount of information and error categories [12], making the data appropriate for analysis and intervention at both individual and systemic levels. Different types of errors necessitate distinct intervention forms, which should help reduce errors in healthcare practices and provide relevant feedback, as illustrated in Figure 1.

Fig 1. *The course of medical error processing with respect to optimizing the healthcare system safety.*

Certain error classification systems can overwhelm healthcare providers, leading to potential underreporting or improper categorization. This may further complicate the analysis of such reports [13].

Medical error taxonomies can be either generic or domain-specific. Generic taxonomies, such as Rasmusen's 1987 classification of errors into skill-, rule-, and knowledge-based types (SRK) [14], offer broad terminology to encompass various fields. Similar is Reason's 1990 model, which builds upon Rasmusen's work with the Generic Error-Modelling System (GEMS). GEMS integrates diverse error mechanisms such as slips, lapses, and mistakes into the SRK framework [15]. Another generic taxonomy is the Joint Commission on Accreditation of Healthcare Organizations (JCAHO) Patient Safety Event Taxonomy (2005), which categorizes reporting systems into five main classifications: impact, type, domain, cause, and prevention and mitigation [16]. However, while such generic taxonomies cover a wide range of domains, their generalization may limit their effectiveness in supporting patient safety recommendations within specific fields [17, 18].

Conversely, domain-specific medical error taxonomies, which contain terms unique to their respective domains, display enhanced reliability due to the consistent terminology used in both taxonomies and incident reports. These taxonomies focus on specific medical areas like primary care [19, 20, 21] or surgery [22, 23]. Examples can be seen in emergency care and

intensive medicine [24, 25, 26, 27]. These taxonomies consider actual patient safety incidents, analyze them, and either create a new taxonomy or modify an existing one to suit the practical needs of patient-centered care in the fast-paced settings of emergency departments and Invensive Care Units. However, the categorizations in these domain-specific taxonomies may pose challenges to information sharing across different domains [28].

A thorough grasp of error taxonomy can be enhanced through structured education and training on medical errors. Regrettably, many existing healthcare curricula might not adequately teach a systematic approach to handling such errors as learning opportunities. By instructing healthcare providers on effective error management, we can foster a safety culture centered on highlighting challenges and executing feasible solutions rather than resorting to blame and penalties in the workplace [29].

The approach to handling medical errors varies between clinical practice and the educational environment. In clinical practice, the focus is on minimizing errors through reliable systems; in education, errors are viewed as valuable learning opportunities. There is an ongoing debate about which error taxonomy framework, clinical practice or education, is more effective.

An educational view is incorporated into simulation-based education. Simulation is a technique that enhances or replaces real patient experiences with deliberately designed scenarios. This approach mimics certain aspects of reality through thorough interaction [30]. By using simulation, educators can link knowledge and the natural environment without endangering patients [31]. Learning in a simulated setting promotes error management strategies and discussion of errors within teams [32]. This fosters communication and collective cognition among team members, key elements in preventing medical errors that could potentially harm a patient [33].

Existing research has not fully explored or applied a medical error taxonomy specifically designed for simulation-based healthcare training in anesthesia, intensive care, and emergency medicine. Such a detailed approach is necessary to identify the root causes of medical errors and improve patient safety and care quality. Our study aims to address this significant gap. We offer unique insights into effective error management strategies in simulation-based education. We assess various taxonomies for categorizing medical errors in this context, debating whether to use a generic or domain-specific taxonomy. This debate seeks to build a framework that not only meets the immediate needs of simulation-based education in these

critical care disciplines but also emphasizes the need for the correct taxonomy to effectively comprehend and reduce medical errors.

2. Methods

We have conducted a review of the literature, leveraging the comprehensive work previously accomplished by Taib [12]. His work critically analyzed medical error taxonomies before 2008. Using his methodology, we assessed how these taxonomies evolved over the past few years, specifying the period between 2009 and 2022. Taib's original paper scrutinized 26 medical error taxonomies from a human factor perspective. It examined whether they encompassed a classification of systematic medical error factors and were founded on theoretical concepts. Using the keywords identical to Taib's original work, we searched Medline, Embase, and PubMed. These terms included patient safety, medical error, taxonomy, and classification. To cross-verify the literature review results, we had two independent researchers check for concordance.

In our literature review, we found no medical error taxonomy specifically for simulation-based healthcare education. This lack presents a knowledge gap. A tailored medical error taxonomy for such education could enable in-depth analysis of prevalent errors across various clinical scenarios. This would aid the design of scenarios mirroring the intricacies of actual healthcare environments, thereby improving the effectiveness of simulation-based healthcare education.

We further explored the recognized knowledge gap by comparing two established medical error taxonomies in the environment of our simulation center. The first one is Reason's GEMS [15]. A potential shortcoming of this system could be its limited direct applicability to healthcare. The classifications are abstract and can be applied universally, as evidenced by the scheme and error description depicted in Figures 2A and 2B.

Fig 2. *(A) Reason's classification: error categories. Adapted from [15]. (B) Reason's classification: definitions.*

Round and his colleagues proposed a second, practical taxonomy in education sessions, which is commonly referred to as the "deadly ten errors" [34]. This taxonomy, illustrated in Figure 3, offers clinical concepts for comprehending the origins of medical errors. It also promotes teaching strategies for error avoidance and is widely implemented in medical education.

Fig 3. *Round's classification: descriptions.*

We carried out a survey with postgraduate participants in high-fidelity simulation courses at Masaryk University's Simulation Centre (SIMU) in Brno, Czech Republic, between March 1, 2021, and October 27, 2022. All participants provided informed consent, which was introduced at the start of the survey. The collected data were anonymized, and the study did not involve any interventions. The study followed all local laws and institutional standards.

During the pre-course e-learning session for the simulation course, both participants and tutors were taught the concept of medical errors, specifically the various types in both taxonomies. The questionnaire, available in Appendix A, provided definitions for each type of medical error as it was presented.

In our survey, we inquired about the prevalence of medical errors. Specifically, participants were asked to indicate whether they observed any particular types of medical errors during a simulation course. This query allowed for binary "yes" or "no" responses. Subsequently, participants were asked to assess the regularity of specific types of medical errors in their daily practice. The survey was designed to make a connection between simulated and actual medical practice. Participants were also provided with an opportunity to detail any particular medical errors that arose during the simulation scenario and to relay their own experiences with medical errors in a clinical setting.

3. Statistics and Results

We gathered data from 124 participants and 13 tutors regarding the incidence of medical errors during the simulation, as well as their rankings of the various types of medical errors encountered in their daily practice. Figure 4 details the process of data preprocessing and analysis.

Fig 4. *Flowchart illustrating the methodology for examining medical error associations. The methodology involved collecting data from 124 participants and 13 tutors, exploring the relationship between medical error classifications using Kendall's tau correlation coefficient, and identifying significant associations with thresholding. Data cleaning, imputation of missing values, and linear rescaling of the resulting data matrix (105 × 15) were performed, followed by analysis to identify the most common types of medical errors and their associations with different classifications.*

We conducted a further analysis of the occurrence matrix data by determining the relative frequencies of medical errors. Figure 5 presents a neatly organized, stacked bar chart

illustrating the distribution of medical errors recorded during the simulation, as reported by both participants and tutors.

Fig 5. *Relative frequency of medical errors observed during the simulation sessions as reported by participants and tutors. The bar charts distinguish and compare two distinct medical error classifications – the Reason's and Round's taxonomies. The x-axis denotes the categories of medical errors, revealing the most and least frequent types of errors within each taxonomy. RBM – Rule-Based Mistakes; KBM – Knowledge-Based Mistakes*

Figure 5 illustrates that, according to Reason's classification, knowledge-based mistakes and lapses were the most common errors observed during the simulation sessions. Round's classification also recorded frequent instances of communication breakdowns and poor team performance. In contrast, the least common errors under Reason's classification were violations, and under Round's classification, sloth. These errors were relatively rare in comparison to other types of medical errors.

We conducted an additional step to understand the ranking of medical errors reported by both tutors and participants. This involved an analysis of their ranking matrices. The findings from this analysis are displayed as a boxplot in Figure 6.

Fig 6. *Boxplot showing participants' and tutors' rankings of medical errors. The y-axis represents the ranking, while the x-axis displays the categories of medical errors. The order of medical mistakes on the x-axis corresponds to the sorted frequencies, as shown in Figure 5.*
RBM – Rule-Based Mistakes; KBM – Knowledge-Based Mistakes.

Upon examining Figure 6, we can see that the patterns depicted in the boxplots for both participants and tutors are strikingly alike. This implies that there is not a significant difference in the rankings given by both groups.

We utilized Kendall's tau correlation coefficient to investigate the relationship between the classifications of medical errors by Reason and Round. Kendall's tau is a nonparametric association measure between two ranked variables, which is suitable for categorized data, like the ranked error frequencies in our study. It is ideal since Kendall's tau makes no assumptions about data distribution and depends solely on the observation order, not their magnitude. This implies that Kendall's tau can identify variable associations, even in a non-linear relationship and remains resistant to outliers.

We executed a thresholding process on the Kendall tau matrix, utilizing a p-value of under 0.1 and a correlation coefficient (tau) absolute value exceeding 0.1 as thresholds. This step helped us spot significant links between various medical error classifications according to frequency rankings by participants and tutors.

Our analysis identified six significant relationships between Reason's ten error types and Round's five error types. We found two associations among the participants and four among the tutors, as shown in Table 1.

TABLE 1. DETECTED CORRELATIONS BETWEEN THE TWO PRESENTED MEDICAL ERROR CLASSIFICATIONS: REASON'S AND ROUND'S TAXONOMIES.

Data from Participants/Tutors	Correlation coefficient	*p*-value	Reason's vs Round's classification
Participants	0.157	0.044 *	Lapses vs. Playing the Odds
Participants	-0.138	0.076	RBM vs. Ignorance
Tutors	0.493	0.047 *	KBM vs Lack of Skill
Tutors	0.482	0.048 *	Lapses vs. Sloth
Tutors	-0.472	0.050	KBM vs. Competence
Tutors	-0.468	0.074	Slips vs Teamwork

RBM – Rule-Based Mistakes; KBM – Knowledge-Based Mistakes
An asterisk (*) next to a *p*-value indicates a statistically significant result.

4. Discussion

While we have been unable to locate and confirm a perfect medical error classification devoted to simulation-based healthcare education, our data provides a fresh look at medical errors in simulation and practice. Our survey reaffirms that the majority of errors stem from inadequate communication and teamwork.

This study examined the correlation between various categories of medical errors based on their reported frequency from participants and tutors in our simulation postgraduate courses. With the use of Kendall's tau, we assessed the intensity and direction of this correlation without assuming any distribution or linearity of the ranked variables. Upon thresholding Kendall's tau matrix, significant connections between the two medical error classifications were found.

Our study results led us to propose interpretations and implementations in our simulation environment that address the correlations between the two classifications presented: Reason's (generic) and Round's (used in medical education), as displayed in Figure 7. Using a single classification could potentially overlook specific medical errors during simulation scenarios.

Fig 7. *Identified correlations between Reason's and Round's classification proposed an interpretation and a possible training modality in a simulation environment.*

Simulation training aims to reflect authentic clinical scenarios, ideally identifying and addressing medical mistakes that could harm patients in a real-life clinical setting. As such, instructors should highlight these errors via "red flags" during debriefing sessions. These errors are vital considerations during the analysis of simulation scenarios. We suggest a pilot simulation course composed of scenarios focusing on Simulation Training Modalities, as shown in Figure 7 from the determined correlation between the proposed medical error taxonomies. This course could offer further insights into the most suitable medical error taxonomy for simulation-based education in healthcare. Notably, the ideal taxonomy could be a blend of the two proposed medical error taxonomies.

While we have yet to discover and validate an optimal medical error classification tailored to simulation-based healthcare education, our data provide fresh insights into medical errors in both simulation and practice. Our survey substantiates that the majority of errors are caused by inadequate communication and less than satisfactory teamwork [35].

Limitations

While a comprehensive literature review was undertaken, it is possible that some pertinent medical error taxonomies were not discovered. To objectively estimate the frequency and types of medical errors, various methods can be used, including analysis of reporting system data [37], the possible application of machine learning [13], patient chart reviews [3] , and direct observation of patient care[38]. Yet, numerous factors influence the reporting of errors and the completeness of patient chart documentation. Providers may refrain from reporting errors due to workload, fear of litigation, complexity of the reporting system, or a punitive work culture.

Our survey data reveals the personal experiences of participants and tutors involved in our simulation courses, often marking their initial engagement with medical error theory.

However, their approach to handling and reporting medical errors may not reflect that of the broader medical community. This divergence could be due to a safe learning environment and adoption of an overall approach towards errors as a learning opportunity.

The authors recognize the need for objective tools in evaluating medical error occurrences. This study, however, strives to connect medical error classification in both practical and simulated environments using a survey. The authors plan on creating and validating objective tools to identify and classify medical errors using a blend of suitable medical error taxonomies observed in healthcare simulation.

5. Conclusion

Our research indicates that there is presently no error classification tailored explicitly for simulation-based healthcare education. The ideal classification would be simple, universally applicable, and focused on the errors that occur most frequently in simulation scenarios. We propose that merging existing classifications instead of generating new ones, would offer better usability for simulation professionals.

The discussion regarding the use of a generic versus a domain-specific medical error taxonomy persists. A generic classification covers a wide range of situations, while a domain-specific one might be more comprehensible and applicable for healthcare professionals, possibly leading to increased medical error reporting.

Our analysis reveals a substantial relationship between various classes of medical errors, indicating their interrelation. By addressing one type of error, others may be positively affected. Furthermore, our study highlights the importance of healthcare simulation as an effective tool for researching, managing medical errors, and enhancing overall performance.

This study uniquely approaches healthcare simulation education by aiming to meet predefined learning outcomes and identifying and addressing ongoing medical errors to enhance patient safety.

Acknowledgments

This research was partially supported by the Specific University Research grant provided to Masaryk University by the Ministry of Education of the Czech Republic (MUNI/A/1595/2023, MUNI/A/1551/2023) and also in part supported by the Ministry of Health of the Czech Republic (FNBr, 65269705). We thank Mr. Radomír Beneš and the

American Manuscript Editors for the English language editing that greatly improved the quality of this manuscript.

References

1. Weingart SN. Epidemiology of medical error. BMJ. 2000;320(7237):774–7.

2. Study Suggests Medical Errors Now Third Leading Cause of Death in the U.S. - 05/03/2016 [Internet]. [cited 22. May 2023]. Available from: https://www.hopkinsmedicine.org/news/media/releases/study_suggests_medical_errors_now_third_leading_cause_of_death_in_the_us

3. Brennan TA, Leape LL, Laird NM, Hebert L, Localio AR, Lawthers AG, et al. Incidence of adverse events and negligence in hospitalized patients: results of the Harvard Medical Practice Study I. N Engl J Med. 1991;324(6):370–6.

4. Wilson RM, Runciman WB, Gibberd RW, Harrison BT, Newby L, Hamilton JD. The Quality in Australian Health Care Study. Medical Journal of Australia. 1995;163(9):458–71.

5. Dhingra DN. WHO Flagship "A Decade of Patient Safety 2020-2030". Patient Safety.

6. Lipsitz LA. Understanding health care as a complex system: the foundation for unintended consequences. JAMA. 2012;308(3):243.

7. Carayon P. Human factors of complex sociotechnical systems. Applied Ergonomics. 2006;37(4):525–35.

8. Kirwan B. Human error identification in human reliability assessment. Part 1: Overview of approaches. Applied Ergonomics. 1992;23(5):299–318.

9. The Difference Between Classification & Taxonomy and Why It Matters | Bounteous [Internet]. [cited 1. March 2024]. Available from: https://www.bounteous.com/insights/2020/11/18/difference-between-classification-taxonomy

10. Donaldson SLJ, Fletcher MG. The WHO World Alliance for Patient Safety: towards the years of living less dangerously. Medical Journal of Australia.2006;184(S10). Dostupné z: https://onlinelibrary.wiley.com/doi/abs/10.5694/j.1326-5377.2006.tb00367.x

11. WHO_IER_PSP_2010.2_eng.pdf [Internet]. [cited 19. červen 2023]. Available from: https://apps.who.int/iris/bitstream/handle/10665/70882/WHO_IER_PSP_2010.2_eng.pdf

12. Taib IA, McIntosh AS, Caponecchia C, Baysari MT. A review of medical error taxonomies: A human factors perspective. Safety Science. 2011;49(5):607–15.

13. Fong A, Behzad S, Pruitt Z, Ratwani RM. A machine learning approach to reclassifying miscellaneous patient safety event reports. J Patient Saf. 2021;17(8):e829–33.

14. Rasmussen J. Information Processing and Human-Machine Interaction. An Approach to Cognitive Engineering. New York: North-Holland; 1987. (North-Holland Series in System Science and Engineering, 12).

15. Human Error [Internet]. [cited 21. February 2024]. Available from: https://books.google.com/books/about/Human_Error.html?id=WJL8NZc8lZ8C

16. Chang A. The JCAHO patient safety event taxonomy: a standardized terminology and classification schema for near misses and adverse events. International Journal for Quality in Health Care. 2005;17(2):95–105.

17. Astion ML, Shojania KG, Hamill TR, Kim S, Ng VL. Classifying laboratory incident reports to identify problems that jeopardize patient safety. Am J Clin Pathol. 2003;120(1):18–26.

18. Shah RK, Kentala E, Healy GB, Roberson DW. Classification and consequences of errors in otolaryngology. The Laryngoscope. 2004;114(8):1322–35.

19. Amalberti R, Brami J. ‘Tempos’ management in primary care: a key factor for classifying adverse events, and improving quality and safety. BMJ Qual Saf. 2012;21(9):729–36.

20. Buetow S, Kiata L, Liew T, Kenealy T, Dovey S, Elwyn G. Patient error: a preliminary taxonomy. The Annals of Family Medicine. 2009;7(3):223–31.

21. Carson-Stevens A, Hibbert P, Williams H, Evans HP, Cooper A, Rees P, et al. Characterising the nature of primary care patient safety incident reports in the England and Wales National Reporting and Learning System: a mixed-methods agenda-setting study for general practice. Health Serv Deliv Res. 2016;4(27):1–76.

22. Suliburk JW, Buck QM, Pirko CJ, Massarweh NN, Barshes NR, Singh H, et al. Analysis of human performance deficiencies associated with surgical adverse events. JAMA Netw Open. 2019;2(7):e198067.

23. Stone A, Jiang ST, Stahl MC, Yang CJ, Smith RV, Mehta V. Development and interrater agreement of a novel classification system combining medical and surgical adverse event reporting. JAMA Otolaryngol Head Neck Surg. 2023;149(5):424.

24. Griffey RT, Schneider RM, Todorov AA, Yaeger L, Sharp BR, Vrablik MC, et al. Critical review, development, and testing of a taxonomy for adverse events and near misses in the emergency department. Acad Emerg Med. 2019;26(6):670-679.

25. Jepson ZK, Darling CE, Kotkowski KA, Bird SB, Arce MW, Volturo GA, et al. Emergency department patient safety incident characterization: an observational analysis of the findings of a standardized peer review process. BMC Emerg Med. 2014;14(1):20.

26. Webman RB, Fritzeen JL, Yang J, Ye GF, Mullan PC, Qureshi FG, et al. Classification and team response to nonroutine events occurring during pediatric trauma resuscitation. Journal of Trauma and Acute Care Surgery. 2016;81(4):666–73.

27. ELMeneza S, AbuShady M. Anonymous reporting of medical errors from The Egyptian Neonatal Safety Training Network. Pediatrics & Neonatology. 2020;61(1):31–5.

28. Taib IA, McIntosh AS, Caponecchia C, Baysari MT. Comparing the usability and reliability of a generic and a domain-specific medical error taxonomy. Safety Science. 2012;50(9):1801–5.

29. Rodziewicz TL, Houseman B, Hipskind JE. Medical Error Reduction and Prevention. In: StatPearls [Internet]. Treasure Island (FL): StatPearls Publishing; 2023 [cited 19. June 2023]. Available from: http://www.ncbi.nlm.nih.gov/books/NBK499956/

30. Gaba DM. The future vision of simulation in health care. Quality and Safety in Health Care. n 2004;13(suppl_1):i2–10.

31. MultiBrief: The role of simulation in the reduction of medical errors [Internet]. [cited 19. June 2023]. Available from: http://exclusive.multibriefs.com/content/the-role-of-simulation-in-the-reduction-of-medical-errors/medical-allied-healthcare

32. D'Angelo AL, Kchir H. Error Management Training in Medical Simulation. In: StatPearls [Internet]. Treasure Island (FL): StatPearls Publishing; 2023 [cited 19. June 2023]. Available from: http://www.ncbi.nlm.nih.gov/books/NBK546709/

33. Østergaard D, Dieckmann P, Lippert A. Simulation and CRM. Best Practice & Research Clinical Anaesthesiology. 2011;25(2):239–49.

34. Vaughan S, Bate T, Round J. Must we get it wrong again? A simple intervention to reduce medical error. Trends in Anaesthesia and Critical Care. 2012;2(3):104–8.

35. Street RL, Petrocelli JV, Amroze A, Bergelt C, Murphy M, Wieting JM, et al. How communication "failed" or "saved the day": counterfactual accounts of medical errors. Journal of Patient Experience. 2020;7(6):1247–54.

36. Wolf ZR, Hughes RG. Error Reporting and Disclosure. In: Hughes RG, editor. Patient Safety and Quality: An Evidence-Based Handbook for Nurses [Internet]. Rockville (MD): Agency for Healthcare Research and Quality (US); 2008 [cited 30. June 2023]. (Advances in Patient Safety). Available from: http://www.ncbi.nlm.nih.gov/books/NBK2652/

37. Goekcimen K, Schwendimann R, Pfeiffer Y, Mohr G, Jaeger C, Mueller S. Addressing patient safety hazards using critical incident reporting in hospitals: a systematic review. J Patient Saf. 2023;19(1):e1–8.

38. Donchin Y, Gopher D, Olin M, Badihi Y, Biesky MR, Sprung CL, et al. A look into the nature and causes of human errors in the intensive care unit: Critical Care Medicine. 1995;23(2):294–300.

APPENDIX A

To err is human; people will continue to make mistakes, and medicine is no exception. However, making mistakes in the simulation center within a safe learning environment is certainly better than in clinical practice. In order to speak the same language when it comes to the theory of errors and be able to properly understand, describe, and evaluate mistakes, it is necessary to first be able to name and categorize them. Your answer will be anonymous. The research team will not identify you in any reports using the information in this questionnaire. By submitting a questionnaire, you agree that your comments will follow as anonymous citations in publications related to this research. Therefore, I kindly ask you to fill it out, which should take no more than 5 minutes. Thank you very much for your cooperation, and I wish you much success in your professional and personal life.

Q1. What types of medical errors do you believe occur in everyday practice?

Rate from 1 (most frequent) to 5 (least frequent), using each number only once:

_ Slips: action-related mistakes, “autopilot”
_ Lapses: memory-based mistakes, we forget what we planned to do or in what order we intended to do it
_ Rule-based mistake: misapplication of a good rule or the failure to apply a good rule, usually because we are not aware of its contraindications or application of a bad rule
_ knowledge-based mistake: related to any type of knowledge, general, specific, or expert
_ Violation: act knowingly incurs a risk

Q2. Have you noticed any medical error(s) during today´s course?

If yes, please mark which one(s) (multiple answers possible).

□ Slips
□ Lapses
□ Rule-based mistake
□ Knowledge-based mistake
□ Violation

Q3. Please describe this error/these errors briefly:

Q4. What types of medical errors do you believe occur in everyday practice?

Rate from 1 (most frequent) to 5 (least frequent), using each number only once:

_ **Sloth:** not doing what you should the effort required or (perceived) is inadequate reward

_**Fixation:** particular diagnosis or analysis is firmly held onto, despite evidence against it, confirmation bias

_ **Communication breakdown:** paramount information is not released or does not reach its destination at the right time

_ **Poor team working team:** members out of their abilities, lack of leadership

_ **Playing the odds:** failure to understand fundamental rules of probability, preferring despite a well-known diagnosis

_ **Bravado:** clinicians work beyond their competence or without adequate supervision

_ **Ignorance:** unconscious incompetence, lack of knowledge

_ **Mis-triage:** over – or underestimation of the seriousness of a situation and lack of prioritization

_ **Lack of skill:** lack of teaching or practice

_ **System error:** unnecessary decision-making steps, multiple distractions, lack of checklist, lack of policies

Q5. Have you noticed any medical error(s) during today´s course?

□ Sloth

□ Fixation

□ Communication breakdown

□ Poor team working team

□ Playing the odds

□ Bravado

□ Ignorance

□ Mis-triage

□ Lack of skill

□ System error

Q6. Please describe this error/these errors briefly:

Fig 1

Click here to access/download;Figure;Fig1.tif

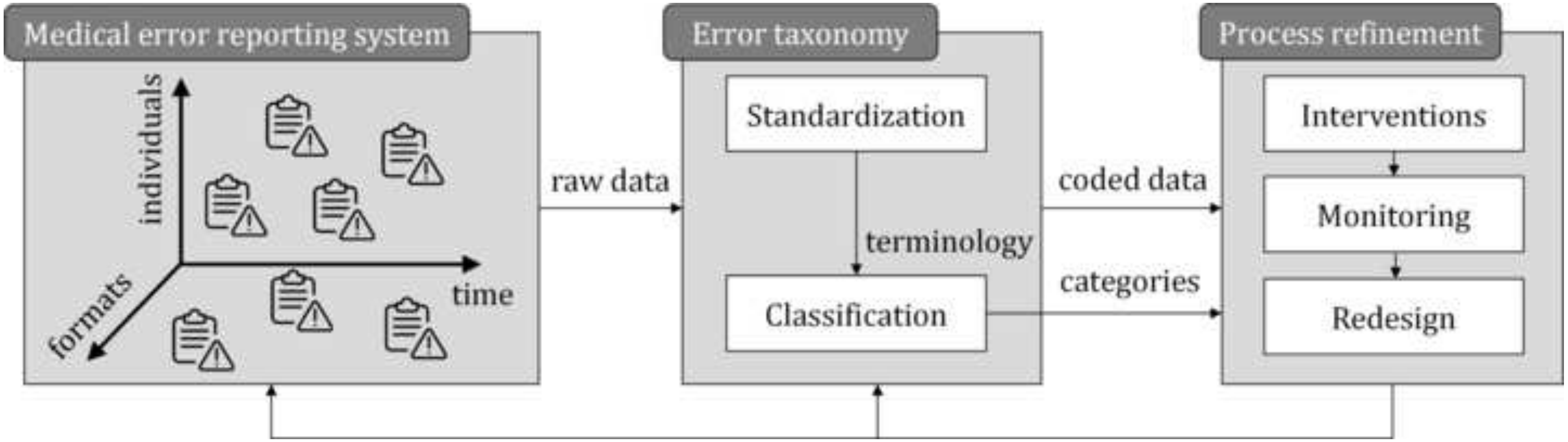

 Click here to access/download;Figure;Fig2ab.tif

A)

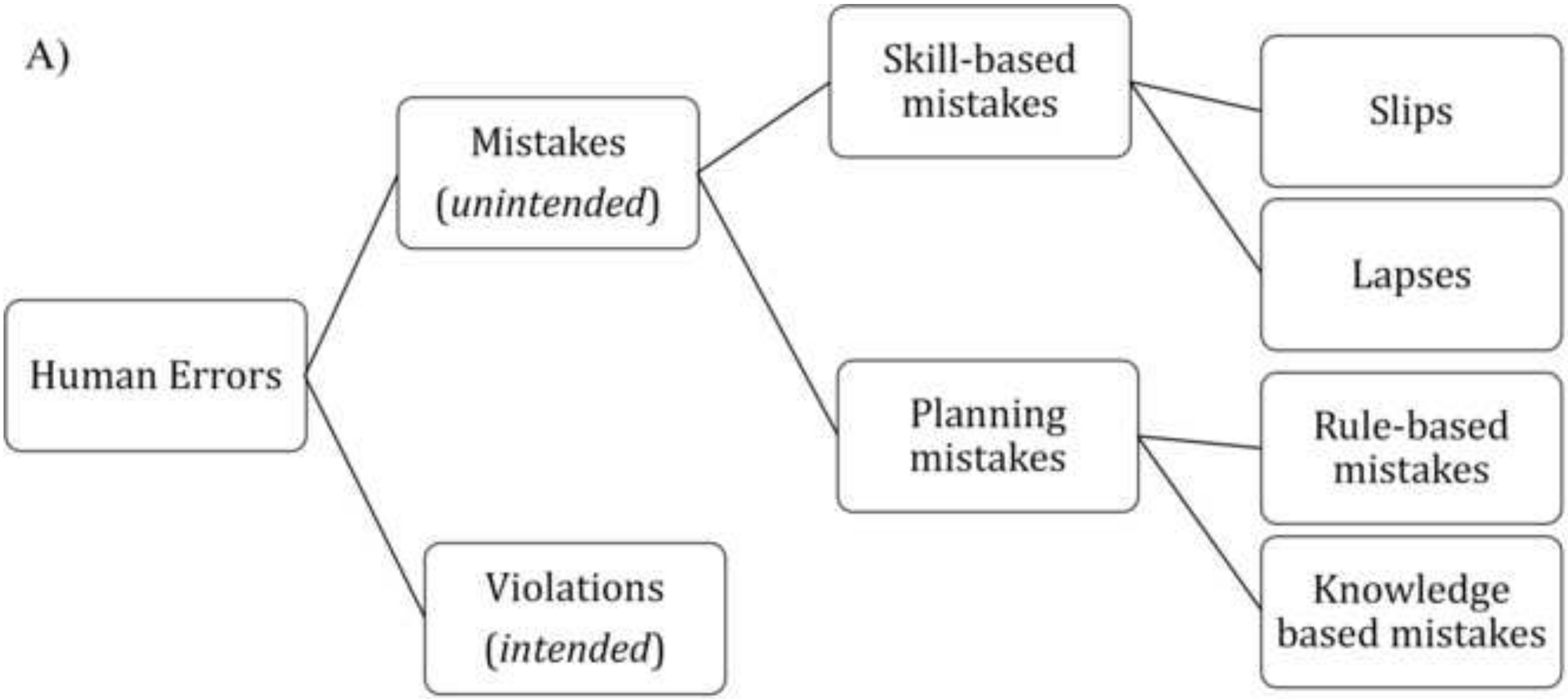

B)

Error Category	Description
Violations	act knowingly incurs a risk
Slips	action-based errors
Lapses	memory-based errors
Rule-based mistakes	a) misapplication of a good rule or the failure to apply a good rule<br>b) application of a bad rule
Knowledge-based mistakes	related to any knowledge, general, specific, or expert

Fig 3

Click here to access/download;Figure;Fig3.tif

Error Category	Description
Sloth	not doing what you should; the effort required or (perceived) is inadequate to the reward
Fixation	particular diagnosis or analysis is firmly held onto, despite any evidence against it; confirmation bias
Communication breakdown	paramount information is not released or does not reach its destination at the right time
Poor team working	team members beyond their capabilities; lack of leadership
Playing the odds	failure to understand the fundamental rules of probability, favouring a well-known diagnosis
Bravado	clinicians work beyond their competence or without adequate supervision
Ignorance	unconscious incompetence; lack of knowledge
Mis-triage	over- or underestimation of the seriousness of a situation and insufficient prioritisation
Lack of skill	lack of teaching or practice
System error	unnecessary decision-making steps; multiple distractions; missing checklist; missing policy

Fig 4

Click here to access/download;Figure;Fig4.tif

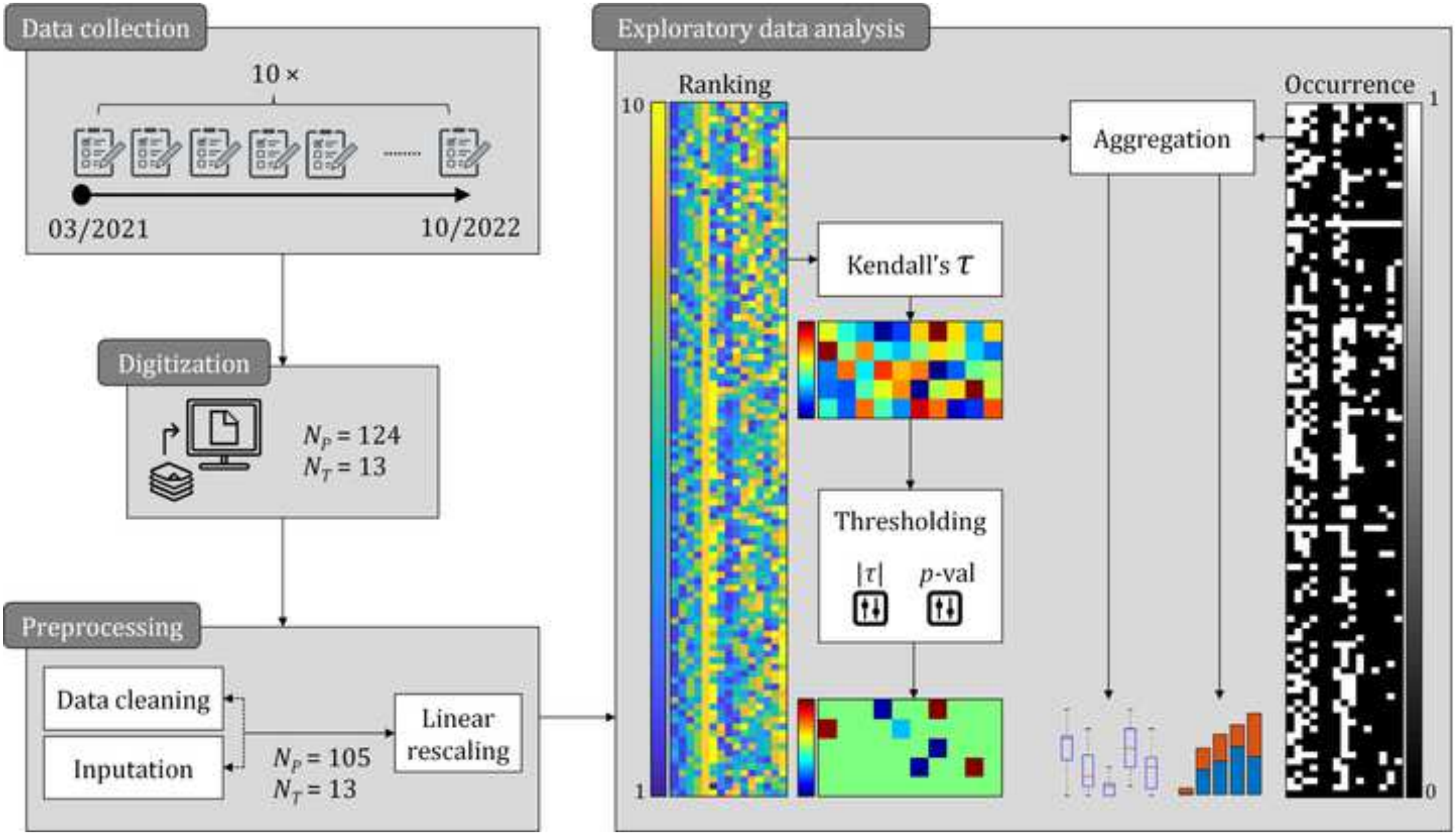

Fig 5

Click here to access/download;Figure;Fig5.tif

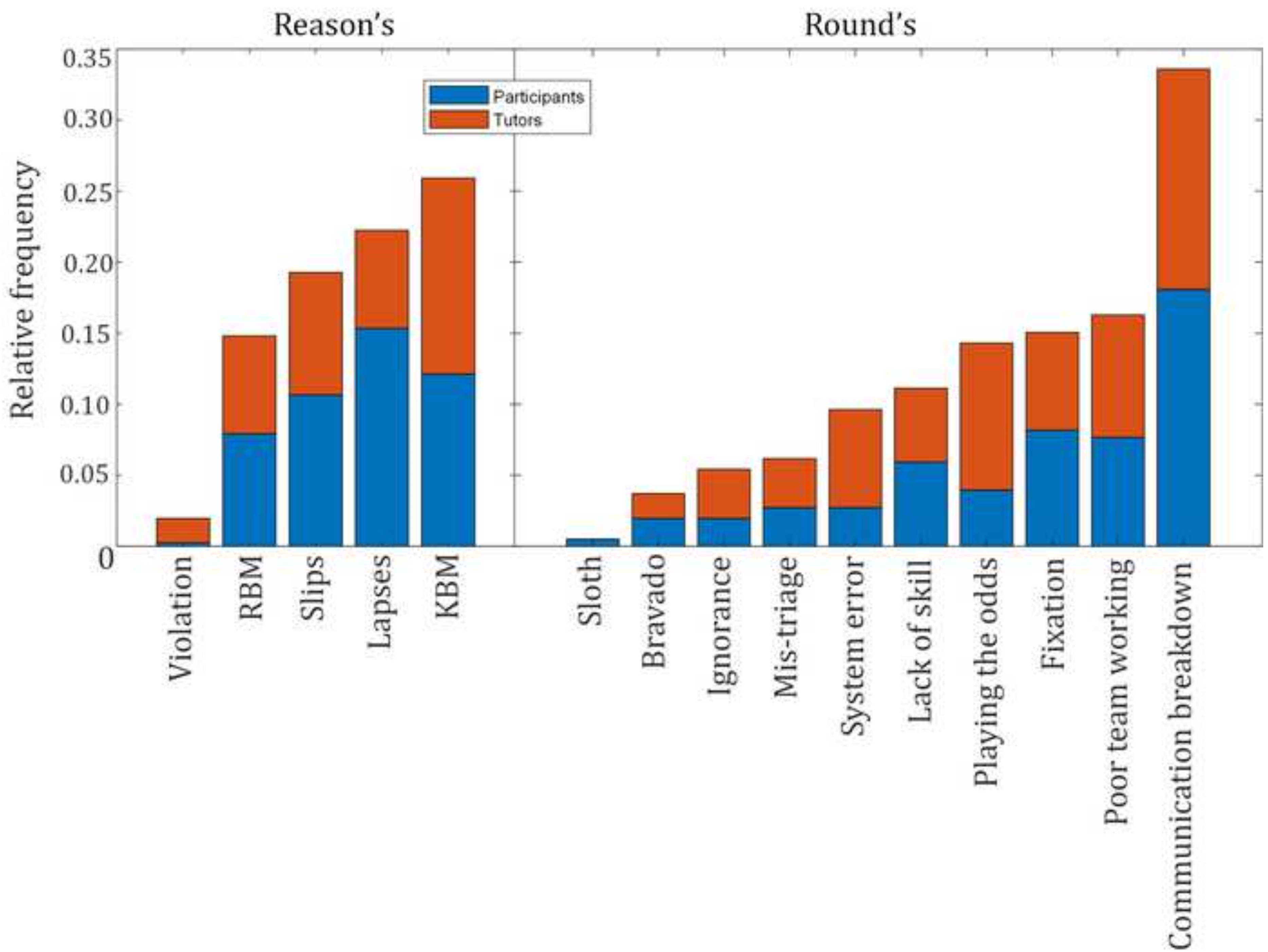

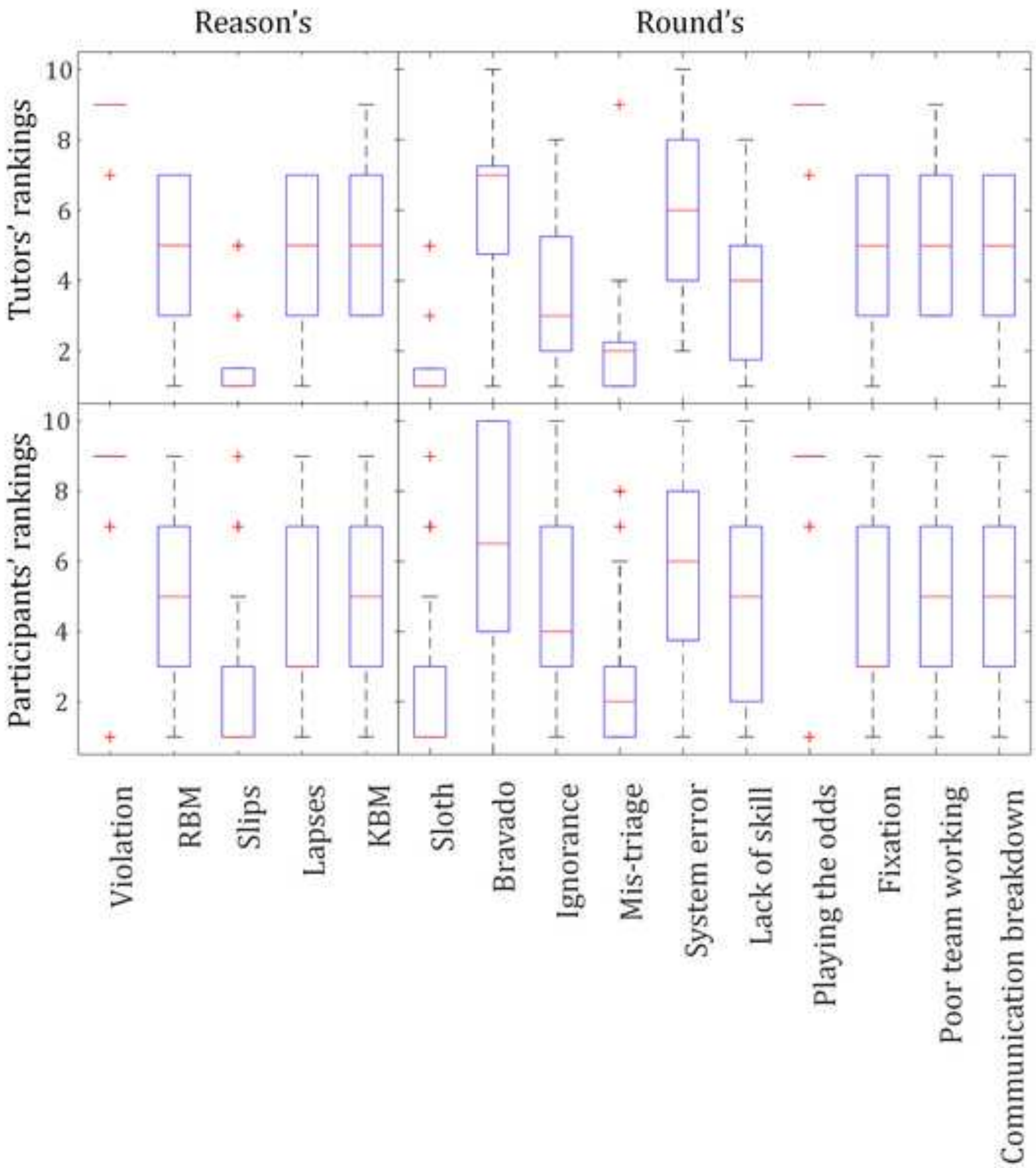
Reason's
Round's
Tutors' rankings
Participants' rankings
10
8
6
4
2
Violation
RBM
Slips
Lapses
KBM
Sloth
Bravado
Ignorance
Mis-triage
System error
Lack of skill
Playing the odds
Fixation
Poor team working
Communication breakdown

Detected Correlation	Proposed Interpretation	Simulation Training Modality
A higher frequency of lapses is associated with a higher frequency of playing-the-odds errors.	Since lapses are associated with memory and often described as "working on autopilot", playing the odds represents the unwillingness to "think out of the box" and approach the case from a different perspective, to admit a diagnosis other than a well-known one –correlating these two errors poses a risk to daily routine practice.	Simulation scenarios based on unusual crises (e.g. malignant hyperthermia during anaesthesia) to get participants focused on non-routine situations.
A higher frequency of rule-based mistakes is associated with a lower frequency of errors caused by ignorance.	RBM means not applying the correct rule or applying the wrong one for the given situation. In both cases, ignorance would suggest not using a rule due to a lack of knowledge or awareness of an existing rule.	Training with cognitive aid of the most important process flows, e.g. CPR guidelines.
A higher frequency of knowledge-based mistakes is associated with a higher frequency of errors caused by the lack of skills.	Both knowledge and skills are required to provide high-quality and safe patient care in the clinical environment.	Knowledge-based mistakes are addressed during the debriefing part. Skills could be practised as a part of a simulation scenario or effectively as skill stations on simulators.
A higher frequency of lapses is associated with a higher frequency of errors caused by a sloth.	Lapses, i.e. errors that occur between the creation and execution of a plan, are among the most frequent in clinical practice. Sloth refers to not doing what we are supposed to due to the effort required or (perceived) inadequate to the expected reward – one of the least frequently occurring errors, according to our data from simulation settings. In other words, "working on autopilot" – the framework in which lapses occur could be related to staying in a comfort zone in a sloth.	A well-executed pre-debriefing, including setting common ground rules and a safe learning environment, usually motivates participants to engage actively, including focusing on the given task. This may serve as an example of quality care in the clinical setting.
A higher frequency of knowledge-based mistakes is associated with a lower frequency of errors caused by bravado.	Ideally, clinicians acquire competence based on their knowledge and expertise. Hence, errors caused by clinicians working beyond their competence or without adequate supervision (bravado) are less likely in more experienced staff and vice versa.	In the simulation, participants are encouraged to call for help early, speak up, and voice their opinion whenever they feel the task is beyond their competence or that an action could lead to patient harm.
According to tutors, a higher frequency of slips is associated with a lower frequency of errors caused by poor teamwork.	A slip is defined as an error caused by a lack of concentration. Good teamwork keeps participants focused on the situation, thus preventing slips.	Situational awareness is one of the most essential aspects of Crisis Resource Management (CRM), often taught during simulation courses.
