# Supplementary material for: Exploring medical error taxonomies and human factors in simulation-based healthcare education
Source: PLoS One. 2025 Jan 17;20(1):e0317128. doi: 10.1371/journal.pone.0317128 (PMC11741583; doi:10.1371/journal.pone.0317128)
Supplement: Attachment — Submitted filename: ExpMedErr-Responses to comments of reviewers.pdf [file pone.0317128.s003.pdf]

Response to Reviewer 1 Comments

General response to all Reviewer1's comments: Dear Reviewer, we would like to express our sincere gratitude for your thorough review of our manuscript and for the numerous valuable comments you provided. We greatly appreciate the time and effort you dedicated to evaluating our work.
However, we would like to bring to your attention that some of your comments, specifically [16, 19, and 20], seem to be less relevant to the focus of our study. These comments pertain to topics related to AI and/or classification algorithms, which are not within the scope of our research. As such, we wonder if there might had been an inadvertent mix-up with another manuscript you were reviewing concurrently.
Below, we have addressed all your suggestions and comments individually.

Point 1: Literature review is shallow.
Response 1: We have conducted a broad literature review on medical error taxonomies based on the review of Dr. Taib (2008), from 2009 to 2022 (later extended to 2024). Initially, the manuscript was submitted as an original research study, but due to the inclusion of an extensive tabulated literature review (featuring a PRISMA diagram), it may not have been immediately clear to some readers why such a detailed review was present in an original research submission. The academic editor specifically recommended resubmitting the paper as an original research paper and removing the review component. We have since revised the manuscript accordingly.
In earlier stages of revision, we explored incorporating additional works (see the list) to reflect emerging trends in medical error taxonomies (Covid-19, AI, etc.). However, to maintain cohesion and align with the study's scope, these were not included in the final version of the manuscript.
Importantly, we have identified a gap in the existing literature – there is currently no clear medical error taxonomy suitable for addressing the specific needs of learners in simulation-based medical education.

- Street RL, Petrocelli JV, Amroze A, et al. How communication "failed" or "saved the day": counterfactual accounts of medical errors. J Patient Exp. 2020;7(6):1247–54.
- Goekcimen K, Schwendimann R, Pfeiffer Y, et al. Addressing patient safety hazards using critical incident reporting in hospitals: a systematic review. J Patient Saf. 2023;19(1):e1–8.
- de Feijter JM, de Grave WS, Muijtjens AM, et al. Overview of medical error in hospitals using incident-reporting systems, patient complaints, and chart review. PLoS ONE. 2012;7(2):e31125.
- Wolf ZR, Hughes RG. Error reporting and disclosure. In: Hughes RG, ed. Patient Safety and Quality: An Evidence-Based Handbook for Nurses. Rockville, MD: AHRQ; 2008.
- Ferrara M, Bertozzi G, Di Fazio N, et al. Risk management and patient safety in the artificial intelligence era: a systematic review. Healthcare. 2024;12(5):549.
- Bohr A, Memarzadeh K. The rise of artificial intelligence in healthcare applications. In: Artificial Intelligence in Healthcare. Elsevier; 2020:25–60.
- Boxley C, Krevat S, Sengupta S, et al. Using community detection techniques to identify themes in COVID-19-related patient safety event reports. J Patient Saf. 2022;18(8):E1196–202.

- Gandhi TK, Singh H. Reducing the risk of diagnostic error in the COVID-19 era. J Hosp Med. 2020;15(6):363–6.
- Rodziewicz TL, Houseman B, Hipskind JE. Medical error reduction and prevention. In: StatPearls. Treasure Island, FL: StatPearls Publishing; 2023.

Point 2: Future scope is missing.
Response 2: We have revised both the Introduction and Discussion sections to clarify the scope of this work. These revisions provide a more detailed explanation of the study's focus and contributions, as well as outlining potential next steps and future research directions.

Point 3: All abbreviations should only be used after their first definition.
Response 3: We carefully reviewed the manuscript and can confirm that all abbreviations were defined at their first occurrence. In the revised version, we also double-checked to ensure that no abbreviations were used without proper definition. We noticed that your comment nr. 21 similarly addresses typographical errors that were not present in the manuscript, which made us wonder if there might be some other issue affecting the document's display or interpretation.

Point 4A: Novelty of the proposed work should be established by comparing the same with comparable work.
Response 4A: We have built up an existing literature search on an extensive review done by Dr Taib (2008) and updated the last version of the manuscript with insights from the last two years, taking into account various existing taxonomies and research done in the field of medical error taxonomies. To our knowledge, we are the first to conduct an exploratory analysis that investigates associations between two widely recognized medical error taxonomies—Reason's and Round's frameworks.

Point 4B: Section should present the methodology algorithmically.
Response 4B: We acknowledge the general nature of this comment and considered the possibility that you may be referring to the Methods section. In response, we have ensured that the methodology is presented with clarity, while maintaining cohesion throughout the manuscript, and taking into account your concern in Point 5.

Point 5: Sections break directly into sub-sections breaking the continuity of the manuscript.
Response 5: We would like to clarify that the text does not include any sub-sections, apart from the customary subsection on Limitations within the Discussion. During the revision process, especially while addressing comments from other reviewers, we worked diligently to enhance the cohesion and flow of the text. We are confident that the current structure provides a clear and well-organized presentation of our research.

Point 6: Sound literature review in tabulated form would be desirable to perform meta-analysis of available work and establish.
Response 6: We appreciate your suggestion. As mentioned in our response to Point 1, we conducted an extensive literature review on medical error taxonomies, which was based on a comprehensive analysis of the work by Dr. Taib. In a previous version of the manuscript, we included a tabulated literature review; however, based on the recommendation of the academic editor, we were encouraged to resubmit the paper as original research. As a result, we focused on the collection, analysis, and interpretation of our own data, and revised the manuscript to align with its aim as an original research article rather than a literature review.

Points 7, 8 & 10. There are areas that require further practical discussion and better linking of the results discussion together. The research methodology is weak. Why did you choose this methodology? The technical and practical discussion, as well as the comparison with recent previous work on this topic, should be thoroughly considered.
The quality of the paper is weak in the technical discussion, and the explanatory results have been discussed before. Also, some parts in the comparison of figures don't make sense. The results are not sufficient, and the conclusion is weak. The references are not sufficient and need a comprehensive update, as well as an update to the sources list until 2024. Major revisions are recommended so that the authors address all of this through examination and validation adequately to meet the standards and strength of the journal.
Both the discussion and practical application and the technical sense are missing. There is no discussion that conveys a true understanding.

Points 7, 8 & 10: We appreciate your feedback and the thorough review of our manuscript. As noted in the other responses, we have made significant revisions, which we believe have strengthened the clarity and depth of both the methodology and the discussion. The methodology used was selected based on its suitability for exploring the associations between two well-established medical error taxonomies, and we have clarified our rationale for this choice in the revised manuscript. Additionally, the discussion has been restructured to more clearly link the results, provide comparison with other findings, and present clearlz strengths and limitations of the study. While we acknowledge your concerns regarding the technical depth and discussion, we believe the revisions have addressed these issues and that our study now provides a solid contribution to the literature.

Point 9: Most sections have written with an AI tools and this missed sense in the information and understanding.
Response 9: We were genuinely surprised by this comment, as it challenges the academic integrity of our work and the standards upheld by our research institution, which is a leading simulation centre in Europe. We would like to clarify that no part of the manuscript was generated using AI tools. The writing was conducted by the research team, with careful attention to clarity and accuracy in the presentation of information. Furthermore, when this manuscript was initially drafted, generative AI tools were not as widely available or integrated into the scientific writing process as they are today. Given that no specific examples or evidence have been provided to substantiate the claim regarding AI-generated sections, we respectfully request further clarification or examples to understand the source of this concern. Additionally, we would like to bring to your attention that certain comments, specifically <16, 19, 20>, seem to be unrelated to the focus of our study and instead pertain to topics such as AI, which are not within the scope of our research. We wonder if there may have been a mix-up with another manuscript under review.

Point 11: The introduction may provide a solid background on the importance of addressing medical errors and patient safety.
Response 11: Thank you for raising the topic. Some papers focused solely on the importance of medical errors and patient safety. Our research dwells on the importance of clear and suitable classification to analyse and sort adequately encountered medical errors in simulation-based medical education.

Point 12: Consider expanding on why existing taxonomies are inadequate

Response 12: We have discussed the advantages and limitations of generic and domain-specific taxonomies. According to a recent Delphi study by Jalali et al., the HFACS for medical errors relies heavily on Reason's GEMS, which is not fully adequate for analyzing medical adverse events. Jalali et al. proposed a novel framework for HFACS, highlighting gaps in current systems. Medical adverse events—harm caused by medical care rather than the patient's condition—are inconsistently researched. Relating these findings to medical errors more broadly, we identified no domain-specific taxonomy suitable for simulation-based healthcare training. Our work contributes to understanding medical error classification systems to support simulation strategies addressing these errors effectively.

Point 13: It is clearly stated that a p-value of under 0.1 and a correlation coefficient (tau) absolute value exceeding 0.1 were used as thresholds. However, the rationale behind choosing these thresholds could be elaborated. Provide a brief explanation for selecting these specific p-value and correlation coefficient thresholds. Example: "A p-value threshold of 0.1 was chosen to capture marginally significant associations, while a tau value exceeding 0.1 was used to identify moderately strong relationships.
Response 13: We agree that further elaboration on the rationale for selecting these thresholds would provide clarity. In response, we have enhanced the explanation in the Statistics and Results section of the manuscript. Specifically, we clarified that the p-value threshold of 0.1 was chosen to increase sensitivity to potentially meaningful associations, which is appropriate for exploratory analyses like ours. This allows us to identify trends that may warrant further investigation. Similarly, the correlation coefficient threshold of $|\tau| > 0.1$ was selected to capture associations with a minimal strength of relationship, ensuring that the identified associations are practically relevant, especially considering the nonparametric nature of our data, where stronger correlations are less common. These changes have been incorporated to better explain the reasoning behind our thresholding process, as reflected in the revised manuscript.

Point 14: Challenges related to error reporting are well-articulated. However, it would be beneficial to propose some strategies for overcoming these barriers.
Response 14: We appreciate this comment. We understand the importance of a well-designed reporting system, and we have included some information based on the literature on the attributes of such a reporting system. Yet again, the topic is very broad, and it is not the primary focus of our study.

Point 15: Suggest methods for mitigating gaps in the literature review and enhancing future taxonomy validation.
Response 15: We greatly appreciate the insightful suggestions regarding future directions for mitigating gaps in the literature and enhancing taxonomy validation. In response, we have expanded the discussion section to outline potential future research. Specifically, we agree that future work could focus on developing a new, comprehensive medical error taxonomy based on our current findings. We envision (in the Discussion) that this taxonomy could be validated in simulation environments using new datasets to ensure its applicability and relevance to simulation-based medical education.

Point 16: The manuscript discusses classifications and their relationships, but there is insufficient analysis on the accuracy and reliability of these classifications. The methodology for evaluating the accuracy of the proposed or merged classifications isn't explicitly detailed.
Response 16: We appreciate the comment, but it seems there may be a misunderstanding regarding the aim of our study. Our research does not propose new classifications; rather, we

explore associations between two established medical error taxonomies (Reason and Round). In our context, the terms 'classification' and 'taxonomy' are used interchangeably, as is common in the field of medical error analysis. The focus of the study is to uncover meaningful relationships between these Reason's and Round's classification systems, rather than to evaluate their accuracy or reliability, as might be the case in algorithm-based classification studies.

Point 17: The accuracy of the proposed classifications needs to be rigorously analyzed. Include metrics such as precision, recall, and F1 score to evaluate the performance of the classifications. Benchmark these against existing classifications to demonstrate their relative effectiveness.
Response 17: As mentioned in our response to Point 16, the aim of our study was not to develop or validate a new classification algorithm, nor were we conducting a performance evaluation that would require metrics like precision, recall, or F1 score. Instead, our study focuses on analyzing associations between pre-existing medical error taxonomies. We hope that, after responding to Point 2, the scope and focus of our study have become clearer.

Point 18: The technical discussion is not currently high-level and lacks depth. Provide detailed descriptions of the methodologies and tools used to develop and implement the classification system in the simulation environment. Include discussions of any technical challenges encountered and how they were addressed.
Response 18: The technical aspects of our study are limited to statistical analysis to identify associations between two medical error taxonomies. We did not develop or implement a new classifier, nor did we encounter technical challenges associated with such development. In our response to Point 13, we have provided a clearer articulation of our methodology and its configurations. We hope this clarifies the nature of the technical aspects of the study.

Point 19: The proposed classification system needs stronger validation. Conduct validation studies in real-world simulation settings and present detailed results and analysis to support the efficacy of the modified taxonomy. Discuss how the findings from these validation studies enhance the credibility of your proposed approach.
Response 19: As our study does not involve the development of a classifier, validation is beyond the scope of our research. Our study aims to explore associations between two established taxonomies, and the identified associations were carefully interpreted by a senior medical educator. We acknowledge the importance of validation in cases where new algorithms/systems are developed, but it is not applicable to our study.

Point 20: Sections of the manuscript involving AI are not well integrated and lack relevance. Clearly explain how AI technologies were utilized in the study, specifying the AI methods used and their role in the classification process. Highlight the impact of AI on the accuracy, efficiency, or reliability of the classification system with specific examples.
Response 20: We did not utilize AI technologies in our study, nor was there any classification process involving AI. The study was based on statistical analysis of the associations between medical error taxonomies. We suspect there may have been some confusion with another manuscript, as AI was not within the scope of our research. We also noticed similar points raised in other comments, such as Points 16-19, which seem to be unrelated to the focus of our study. We hope our responses to these points clarify the nature of our work and help resolve any potential confusion.

Point 21: The paper needs proofreading to correct any typographical, grammatical, or structural errors. Ensure that terms and abbreviations are defined upon first use and employed consistently throughout the manuscript. Address any incomplete or unclear phrases, such as "human limitatUnderstanding."
Response 21: Our manuscript was proofread by professional editors from AmericanManuscriptEditors, and we have provided a certificate to confirm this. The same applies to the current revised version that has been re-submitted for your kind consideration. We have only changed the service and used Editage.com. The specific typographical error you mentioned did not appear in the submitted manuscript. The words "human limitations." on line 42 and "Understanding the nature..." on line 43 had been correctly separated. Could it be that you were using an outdated version of Word or perhaps an alternative Office software, which might be displaying the manuscript incorrectly?

Point 22: Eaborate on how the study's findings will be practically applied in simulation training. Provide specific examples of scenarios or training modules where the insights will be implemented. Detail how "red flags" will be incorporated into debriefing sessions.
Response 22: We aimed to propose simulation training strategies in Figure 7. Examples include the use of cognitive aid and principles of Crisis Resource Management. We aim to include also scenarios of uncommon critical situations (e.g. malignant hyperthermia) to make participants come out of their autopilot settings (where most of the lapses errors are happening) as well as thinking out of the box in the differential diagnosis (in this case examine any otherwise inexplicable increased endCO2-red flag if missed by the participants with potential for catastrophic consequences). The "red flags" would be identified in advance for each scenario and would serve as a reminder to debriefers that this was a gap that must be addressed during the debriefing.

Response to Reviewer 2 Comments

General response to all Reviewer2's comments: Dear reviewer, thank you for you comments on our manuscript. We addressed them carefully and also changed some formal mistakes in the text based on your recommendations.

Point 1: The abstract and the suggested intervention should be clearly mentioned
Also please mention the role of simulation in addressing medical errors in postgraduate courses. A brief mention of the type of postgraduate courses is required.
Response 1: We appreciate your feedback. Information regarding the intervention, including the role of simulation in addressing medical errors, has been included in the abstract and specified in the revised Introduction. We have also clarified that the referenced postgraduate courses are based in Critical Care settings. To improve cohesion, the Introduction and Discussion sections have been significantly revised.

Point 2: Objectives are missing and are not clearly defined explaining the aim and rationale of research. The introduction is also very lengthy and should be made concise.
Response 2: Thank you for this valuable comment.We have revised the abstract and the introduction to clearly state the objectives of our study. Furthermore, we have shortened the introduction, with some of the content being relocated to the discussion section to ensure a more concise presentation.

Point 3: Please mention the type and method of simulation used, whether it was OSCE, who was the assessor and how was the grading done
Response 3: We have clarified in the manuscript that the simulation used was high-fidelity, specifically focusing on postgraduate courses related to Critical Care. The participants were not formally assessed, as the discussion about errors encountered in the scenarios was incorporated into part of the debriefing process.

Point 4: This study does not clearly mention how it addresses the use of strategies employed in simulation based courses to address medical errors in postgraduate education. This is a highly significant factor in terms of patient outcomes adversely affected by medical errors in clinical practice.
Response: Figure 7 illustrates the specific simulation training strategies proposed to address errors identified in both classification systems. The role of simulation in reducing medical errors in postgraduate education is largely based on training in Crisis Resource Management principles, with a strong focus on improving communication practices—areas frequently emphasized during debriefings, even within highly experienced teams. While there is limited data directly linking simulation-based training to improved clinical outcomes, we sought to explore by including questions in our survey about the errors encountered in the simulation and the personal experiences of both tutors and participants regarding errors in clinical practice, aiming to connect the simulation environment with real-world clinical practice.

Point 5: Strengths of the study should also be mentioned. How this study contributes to the existing medical literature.
Response 5: We have significantly revised the Discussion section, which now includes dedicated subsections on the strengths and limitations of the study, as well as a concluding subsection. These revisions highlight how our study contributes to the existing medical literature and provide a clearer and more structured presentation of our findings.

Response to Reviewer 3 Comments

General response to Reviewer3's all comments: Dear reviewer, we sincerely appreciate your constructive feedback and your positive engagement with our manuscript. Your suggestions have been very helpful in improving the clarity and comprehensiveness of our work, and we have carefully considered each of your points.

Point 1: Methodology can be improved by clearly stating the study design of this manuscript in the beginning of the section.
Response1: Thank you for highlighting this important point. We have now added a statement at the beginning of the methodology section, clarifying that our study is an exploratory analysis. We believe this will enhance the reader's understanding of the research design from the outset.

Point 2: As in first glance it appears to be a review. It would be good to clarify the various taxonomies and their usage in both types of medical errors with clarity instead of just a table.
Response 2: We conducted an extensive literature review, including tabular summaries and a PRISMA chart. However, due to the exploratory nature of our manuscript, we were advised by the academic editor to resubmit it as original research, removing the review component. In this major revision, we have ensured that the design of the paper is clear, with the abstract and introduction revised to make our aims and objectives more comprehensible.

Point 3: The number of participants should be stated in methodology section instead of statistical analysis.
Response 3: We have moved the information about the number of participants to the methodology section, ensuring clarity and better organization.

Point 4: The level of the learner should be clearly specified. Is the study conducted on undergraduates, interns or residents?
Response 4: Thank you for pointing this out. The participants in our study were postgraduate resident learners, and we have included this information both in the abstract and in the introduction to provide appropriate context.

Point 5: Discussion should be more detailed and comprehensive, comparing the study results with previous studies.
Response 5: We appreciate your suggestion. The Discussion section has been significantly revised and now includes dedicated subsections on strengths, limitations, and a conclusion. Additionally, we have expanded the discussion of our results, providing comparisons with previous studies.

Point 6: A lot of material elaborated in the limitation's sections can be a part of discussion.
Response 6: Thank you for this observation. In an effort to keep the text coherent and readable, we have maintained a clear separation between the discussion and limitations. However, we did incorporate more detailed information in the limitations section to ensure that the rationale for these limitations is fully explained.

Point 7: Last paragraph of limitations is way forward, it should be separated from limitations
Response 7: We agree with your suggestion. We have moved the last paragraph of the limitations to the conclusion section, where it serves as a basis for outlining future research directions.
